# Microbiological survey and genomic analysis of *Cronobacter sakazakii* strains isolated from US households and retail foods

**DOI:** 10.1128/aem.00700-24

**Published:** 2024-07-02

**Authors:** Mansour Samadpour, Lora Benoit, Sam Myoda, Bada Hans, Cesar Nadala, Seong Hong Kim, Eni Themeli, Ruth Cantera, Truyen Nguyen, Hans Richter

**Affiliations:** 1 IEH Laboratories and Consulting Group Inc., Lake Forest Park, Washington, USA; Universita degli Studi di Napoli Federico II, Portici, Italy

**Keywords:** food safety, infant formula, food pathogen, *Cronobacter*

## Abstract

*Cronobacter *species are opportunistic pathogens that are capable of causing morbidity and mortality, particularly in infants. Although the transmission dynamics involved in *Cronobacter* infections remain largely unknown, contaminated powdered infant formula (PIF) has been linked to 30% of *Cronobacter sakazakii* cases involving invasive illness in infants. As several lines of evidence have implicated the domestic environment in PIF contamination, we undertook a microbiological survey of homes (*N* = 263) across the US. *Cronobacter* spp. and *C. sakazakii* were isolated from 36.1% and 24.7% of US homes, respectively, with higher recovery rates observed for floor and kitchen surfaces. Multi-locus sequence typing indicated that the dominant strain was *C. sakazakii* ST4, the sequence type most commonly associated with neonatal meningitis. For comparison purposes, retail foods (*N* = 4,009) were also surveyed, with the highest contamination frequencies (10.1%–26.3%) seen for nut products, seeds, and grains/baked goods/flours. The sequence type profile of isolates recovered from homes mirrored that of isolates recovered from retail foods, with increased representation of ST1, ST4, ST13, ST17, and ST40. Analysis of 386 whole genomic sequences revealed significant diversity. Redundancies were only observed for isolates recovered from within the same domicile, and there were no identical matches with sequences archived at the NCBI pathogen database. Genes coding for putative virulence and antibiotic resistance factors did not segregate with clinically significant sequence types. Collectively, these findings support the possibility that contamination events occurring within the home should not be overlooked as a contributor to community-onset *Cronobacter* infections.

**IMPORTANCE:**

*Cronobacter sakazakii* is an opportunistic pathogen that can cause significant morbidity and mortality in neonates. Its transmission dynamics are poorly understood, though powered infant formula (PIF) is thought to be the major transmission vehicle. How the PIF becomes contaminated remains unknown. Our survey shows that roughly 1/4 of US homes are contaminated with *Cronobacter sakazakii*, particularly in the kitchen setting. Our analyses suggest that the domestic environment may contribute to contamination of PIF and provides insights into mitigating the risk of transmission.

## INTRODUCTION

*Cronobacter* spp.*,* previously known as *Enterobacter sakazakii*, are gram-negative, typically motile, non-spore-forming bacilli belonging to the Enterbacterales order (1–3). The genus encompasses seven species: *C. sakazakii, C. malonaticus, C. turicensis, C. muytjensii, C. dublinensis, C. universalis*, and *C. condimenti* (3, 4). *Cronobacter* spp. are classified as opportunistic pathogens that are acquired both nosocomially and in the community setting. Most infections are caused by *C. sakazakii*; however, all members of the genus, except for *C. condimenti*, have been linked to clinical cases of infection (5–9). Infections occur in all age groups and can involve multiple anatomical sites, including the urinary tract, skin, respiratory tract, blood, gastrointestinal tract, central nervous system, conjunctiva, and bone (5–11). In adults, infections are generally non-life-threatening and typically occur in the context of underlying health issues or advanced age (9, 11). In contrast, infections in infants less than 2 months of age can manifest as necrotizing enterocolitis (NEC), sepsis, and meningitis, with fatality estimated at 40% (8, 10–14). Although the prevalence of *Cronobacter* infections in infants is relatively low (10, 11), the potential for severe illness as well as lifelong sequelae (15) has earned *Cronobacter* spp. the status of most costly foodborne pathogen in the US, with an estimated per case healthcare cost of 1 million USD (16).

While the transmission dynamics of *Cronobacter* infections are largely unknown, a significant vehicle for transmission in infants is thought to be powdered infant formula (PIF) (10, 17–22). As such, testing of available PIF containers is regularly undertaken in the course of source investigations involving infants. Detection of the organism in sealed, lot-matched containers denotes contamination originating during the manufacturing process and is referred to as intrinsic product contamination. Detection of the organism in previously opened PIF containers can signify extrinsic product contamination, though it does not eliminate the possibility of intrinsic contamination. Outside the US, cases of invasive *Cronobacter* illnesses in infants have been linked to both intrinsically contaminated PIF and previously opened containers of PIF. Since 2004, the majority of non-US cases (85%) have occurred in the hospital setting, primarily as outbreak-associated, epidemiologically linked cases (10). Within the US, the pattern is strikingly different; since 2004, no cases have been linked to intrinsically contaminated PIF (10). Furthermore, the majority of US cases (78%) have primarily occurred in the community setting as sporadic, epidemiologically unlinked cases (10, 20).

To better define the transmission dynamics, the US Center for Disease Control and Prevention (CDC) recently undertook a retrospective analysis of cases involving invasive *Cronobacter* illness in infants. From their analyses of 71 source investigations, contaminated opened PIF containers were identified in 30% (21 of 71) of cases, and contaminated surfaces and/or foods from within the home were identified in 44% (31 of 71) of cases (10). Transmission vehicles identified by these investigations included bottled PIF reconstitution water (10, 13), kitchen sink surfaces (10), vacuum cleaner contents (23), breast pump components (10, 24), pacifiers (10), bottle components (10), and food blenders (10). Additionally, 10% of source-investigated cases involved infants fed exclusively with expressed breastmilk. The clinical isolates in these instances matched environmental isolates recovered from breast milk samples, breast pump kit components, the kitchen sink, and the drying area next to the sink (10, 24–26). Although it is possible that some of these cases may involve intrinsic PIF contamination, these findings point at the possibility that PIF contamination in the US is occurring extrinsically, in either the hospital or home setting.

A number of surveillance studies have assessed the domestic environment as a possible source of infection. Within the US, *Cronobacter* spp. were recovered from 78.5% (51 of 65) of homes sampled in Georgia (27), while a separate study identified *C. sakazakii* in 26.9% (21 of 78) of homes in Tennessee (28). Sites within the domestic environment from which *Cronobacter* spp. have been reproducibly recovered include vacuum cleaner contents, floors, entryways, refrigerators, sinks, kitchen cleaning cloths, and kitchen sponges (27–33). Importantly, the conspicuous presence of *Cronobacter* spp., particularly *C. sakazakii*, in the domestic environment establishes clear opportunities for contamination of food and infant formula, potentiating foodborne illnesses. Therefore, to expand on these earlier, smaller studies, we undertook a large-scale survey of retail foods and households across the US. Isolates collected during the course of this survey underwent WGS analysis and sequence typing to assess the genetic diversity and potential clinical significance. The results of this study extend our understanding of *Cronobacter* transmission dynamics and support the use of mitigation strategies to reduce the risk of infection in the domestic environment. Importantly, the results of this study should be considered both narrowly and broadly, as caregiver awareness of the possible transmission risks arising from the domestic environment may play a crucial role in ensuring both the safety and security of infant formula. In this regard, in 2022, a major PIF manufacturer issued a voluntary recall based on the presumption of intrinsic contamination following four sporadic cases of community-onset invasive *Cronobacter* illness in neonates occurring over the period of September 2021 to February 2022 (34). However, subsequent product testing was unable to provide evidence for intrinsic contamination of PIF as the source of these infant illnesses (34). The recall led to nationwide shortages that limited formula availability for infants generally and for infants with special nutritional requirements over a period of several months (35).

## MATERIALS AND METHODS

### Domestic environment samples

For the period of June 2022 to June 2023, households throughout the US were mailed sampling kits and overnight return packaging with re-freezable ice packs. The sampling kits included MegaSampler Sponges with DE Neutralizing Broth (Weber Scientific, Hamilton, NJ) and sterile sampling bags (Weber Scientific, Hamilton, NJ). Study participants were instructed to surface test bathroom areas (floors, sink, and countertops), kitchen areas (floors, sink, countertops, and sponges), footwear, car mats, entryway floors, and refrigerator shelves using the sponges and to collect floor sweepings or vacuum cleaner contents into the sampling bag. The manufacturer’s instructions for sampling were supplied with the kits. In some instances, study participants collected multiple samples for the same sample category or missed a sample category. In total, 2,810 environmental samples from 263 homes from 36 different states across the continental US were collected and analyzed.

### Food samples

For comparison purposes, a total of 4,009 retail food items purchased across the US were analyzed throughout the period of March 2022 to August 2023. Each food sample tested was from a distinct lot. Food items included grains, milled flours, baked goods, confections, seeds and beans, raw beef, raw chicken, sprouts, nuts, nut butters, candies, snack foods, chocolate, pet food, trail mix, nutritional supplements, botanical products, noodles, pasta, fresh fruits, fresh vegetables, and spices/seasonings. Product descriptions are provided in Table 2.

### Sample enrichment

Food samples (either 25 or 375 g) were enriched by the addition of M1 (GN) medium (either 75 or 1,125 mL, respectively) intended for gram-negative bacteria (Microbiologique, Seattle, WA) as per the manufacturer’s instructions and grown 26 hours t at 42°C. Swabs were placed in 100 mL of 2% peptone-buffered water and grown for 26 hours at 35°C.

### PCR screening

Enrichments were screened for the presence of *Cronobacter* spp. using the Microbiologique *Cronobacter* and *Salmonella* Screening PCR Kit (Microbiologique, Seattle, WA) as per the kit instructions. Enrichment broths from presumptive positive PCR samples were then streaked onto RF medium and Harlequin medium plates to obtain isolated colonies. Confirmation was performed by colony PCR using the *Cronobacter* and *Salmonella* Confirmation PCR Kit (Microbiologique, Seattle, WA) as per the kit instructions.

### Culture confirmation

All presumptive positive enrichment cultures were subjected to culture confirmation using the FDA Bacteriological Analytical Manual (BAM) method (36). Colonies from all culture-confirmed samples were subjected to whole-genome sequencing analysis.

### Whole-genome sequencing and MLST sequence typing

Genomic DNA samples from 415 confirmed isolates were submitted for WGS. Genomic libraries were prepared using the Nextera XT DNA Sample Prep Kit (Illumina, CA), and genome sequencing was performed using the Illumina MiSeq desktop sequencer (Illumina, CA) loaded with a paired-end 2 × 250 cycle MiSeq reagent kit version 3. The raw sequencing reads were quality filtered using the default settings of the “fastp” version 0.20.0 software (37). After sequencing, FastQC v0.11.7 (38) was utilized to perform quality control checks on the raw sequence data. The filtered reads were then assembled using the SPAdes assembler (https://github.com/ablab/spades/tree/v3.15.4). The quality of the genome assemblies was evaluated using Quast version 4.6.3 (39). Any data with metrics below 30 for *R*1 and *R*2, coverage less than 20×, an assembly sequencing length lower than 4 million base pairs or higher than 5 million base pairs, and more than 500 contigs were excluded from the analysis. The resulting *de novo* assemblies were then used for preliminary identification and cross comparisons using the MinHash algorithm implemented in an in-house mash database built using the RefSeq 210 database (40, 41). From the whole-genome assemblies, seven-locus multi-locus sequence typing (MLST) was performed using the SRST2 version 0.2.0 software (https://genomemedicine.biomedcentral.com/articles/10.1186/s13073-014-0090-6). The assembled files were submitted to the TYGS server for genus-/species-level verification (42). This cross-referencing comparison confirmed the speciation of the *Cronobacter* species from the *de novo* assemblies. Sequence types were confirmed using MLST tool available at https://pubmlst.org/. The sequences were then compared against the NCBI Pathogen Detection Database (43). The SNP distances within the SNP cluster groups were compared using the CFSAN SNP pipeline (44). Sequences that satisfied the QC thresholds described above were deposited at the NCBI Pathogen Detection Database. The Biosample IDs for the assembled whole-genome sequences deposited at NCBI are listed in the Supplemental Material section.

### Virulence analysis

Whole-genome sequences of 386 isolates were uploaded into the Bacterial and Viral Bioinformatics Resource Center (BV-BRC) (https://www.bv-brc.org) (45) and interrogated for factors associated with virulence, including secretion systems, adhesion factors, infection/invasion/host resistance factors, toxins, as well as antibiotic resistance enzymes and efflux pumps to ascertain the total number of genomes that contained each factor. In addition, factors associated with infection/invasion/host resistance were further analyzed for percent identity against reference sequences obtained from NCBI to compare genetic distinctions among the five most common sequence types identified in this study, namely ST1, ST4, ST13, ST17, and ST40. Sequences from NCBI included CsakCS931_RS20575 (*Cpa*), CsakCS931_RS08525 (*EfeO*), CsakCS931_RS09290 (*Fha*), CsakCS931_RS17840 (*FkpA*), CsakCS931_RS18715 (*Hfq*), CsakCS931_RS18895 (*HlyIII*), AFK66_RS20815 (*IbeB*), CsakCS931_RS21050 (*IucA*), CsakCS931_RS14245 (*NanR*), CsakCS931_RS15355 (*SodA*), and CsakCS931_RS03030 (*WcaD*). In all but one instance, the sequences used in the queries were specific for *C. sakazakii*. A novel allele was inferred on the basis of ≥0.002% difference in percent identity, and the numbering of alleles was arbitrarily assigned.

### Phylogenetic analysis

Cronobacter MLST allele profiles were derived from https://pubmlst.org/bigsdb?db=pubmlst_cronobacter_seqdef&page=downloadAlleles&scheme_id=1&render=1. A blastn database of the *Cronobacter* MLST alleles was created on a local server using makeblastdb (https://www.ncbi.nlm.nih.gov/books/NBK569841/). MLST sequences were extracted from *de novo* assemblies using a local blastn database and a Python in-house script. The sequences were then concatenated to each sample, as performed by Rachlin et al. (46). Alignment was performed using MAFFT (47), followed by the creation of a phylogenetic tree using maximum likelihood estimation performed using Maga 11 software. The tree was visualized using iTOL version 6 software (https://itol.embl.de/).

## RESULTS

### Occurrence of *Cronobacter* spp. and *Cronobacter sakazakii* in US homes

To survey the domestic environment, samples were collected from a total of 263 homes located in 36 continental US states for the period of June 2022 to June 2023. On average, roughly 11 different samples were collected from each home. Samples were processed as described in the Materials and Methods section. PCR presumptive positive samples (*N* = 516, 382 of which were *C. sakazakii* and 134 of which were non-*sakazakii Cronobacter* spp.) underwent BAM method culture confirmation. Using this approach, 88.7% (339 of 38) of the *C. sakazakii* presumptive positive samples were confirmed positive. Correspondingly, 67.2% (90 of 134) of the non-*sakazakii Cronobacter* spp. presumptive positive isolates were confirmed positive and subsequently speciated through WGS analysis. Of the 263 US homes tested, 36.1% were positive for *Cronobacter* spp., and 24.7% were positive for *C. sakazakii*. As for the other species, 6.5% of homes were positive for *C. turicensis,* 3.8% were positive for *C. malonaticus*, 3.4% were positive for *C. dublinensis*, 2.3% for *C. muytjensii*, and 0.8% were positive for *C. universalis*. Furthermore, 5.3% of homes were positive for two different species. For the 65 US homes that were positive for *C. sakazakii,* the following observations were made: 39 homes were positive for a single isolate, 6 homes were positive for multiple isolates (2–4) belonging to the same sequence type, 11 homes were positive for multiple sequence types (2–7), and 9 were positive for two or more different species including *C. sakazakii* (data not shown). In instances where multiple strains of the same ST were isolated from the same household, the number of SNPs (determined by WGS comparisons) ranged from 0 to 91. A total of 21 homes (8% of total) were positive for ST4, the sequence type associated with neonatal meningitis (13). Correspondingly, 28 homes were positive for one or more non-*sakazakii* species.

Within the home setting, *Cronobacter* spp. were most frequently isolated from entryway floors (22.8%, 13 of 57 samples), vacuum cleaner contents/floor sweepings (18.3%, 70 of 382 samples), and car mats (9.7%, 21 of 217 samples) (Table 1). Intermediate isolation recoveries were observed for kitchen surfaces, including kitchen sponges (7.0%, 5 of 71 samples), kitchen sinks (5.3%, 14 of 265 samples), kitchen floors (3.5%, 10 of 282 samples), kitchen counters (2.8%, 8 of 283 samples), and refrigerator shelves (2.4%, 5 of 210 samples). The lowest isolation rates were observed for bathroom surfaces, which were consistently less than 1%. As with other *Cronobacter* spp., the distribution frequencies of *C. sakazakii* were similar, with the most frequent isolation sites consisting of entryway floors (21.1%, 12 of 57 samples) and vacuum cleaner contents/floor sweepings (12.8%, 49 of 382 samples), though recovery from car mat samples was lower (3.2%, 7 of 217 samples). Similarly, intermediate isolation recovery rates were observed for kitchen sites, including kitchen sinks (3.8%, 10 of 265 samples), kitchen sponges (2.8%, 2 of 71 samples), kitchen floors (2.8%, 8 of 282 samples), kitchen counters (2.1%, 6 of 283 samples), and refrigerator shelves (1.4%, 3 of 210 samples). As with *Cronobacter* spp.*,* the lowest recoveries of *C. sakazakii* were seen for bathroom surfaces.

**TABLE 1:** Recovery of *Cronobacter* spp. from different sites within US homes (*N* = 263)

Domestic environment collection sites^*a*^	No. of samples tested	*Cronobacter* spp. positives no. (%)	*C. sakazakii* positives no. (%)	*C. turicensis* positives no. (%)	*C. malonaticus* positives no. (%)	*C. dublinensis* positives no. (%)	*C. muytjensii* positives no. (%)	*C. universalis* positives no. (%)
Entryway floor	57	13 (22.8)	12 (21.1)	0 (0)	1 (1.8)	0 (0)	0 (0)	0 (0)
Sweeping/vacuum dust	382	70 (18.3)	49 (12.8)	12 (3.1)	2 (0.5)	5 (1.3)	2 (0.5)	0 (0)
Car mats	217	21 (9.7)	7 (3.2)	4 (1.8)	5 (2.3)	2 (0.9)	3 (1.4)	0 (0)
Kitchen sponge	71	5 (7.0)	2 (2.8)	0 (0)	2 (2.8)	0 (0)	0 (0)	1 (1.4)
Kitchen sink	265	14 (5.3)	10 (3.8)	0 (0)	2 (0.8)	0 (0)	1 (0.4)	1 (0.4)
Footwear	547	27 (4.9)	22 (4.0)	3 (0.5)	1 (0.2)	1 (0.2)	0 (0)	0 (0)
Kitchen floor	282	10 (3.5)	8 (2.8)	1 (0.4)	0 (0)	1 (0.4)	0 (0)	0 (0)
Kitchen counter	283	8 (2.8)	6 (2.1)	0 (0)	1 (0.4)	1 (0.4)	0 (0)	0 (0)
Refrigerator shelves	210	5 (2.4)	3 (1.4)	0 (0)	1 (0.5)	0 (0)	1 (0.5)	0 (0)
Bathroom counter	130	1 (0.8)	1 (0.8)	0 (0)	0 (0)	0 (0)	0 (0)	0 (0)
Bathroom floor	144	1 (0.7)	1 (0.7)	0 (0)	0 (0)	0 (0)	0 (0)	0 (0)
Bathroom sink	222	1 (0.5)	1 (0.5)	0 (0)	0 (0)	0 (0)	0 (0)	0 (0)
Total	2,810	176 (6.3)	122 (4.3)	20 (0.7)	15 (0.5)	10 (0.4)	7 (0.2)	2 (0.1)

^*a*^ Culture-confirmed positive samples are expressed as the number of positive sites and parenthetically as the percentage of total samples in each category.

### Occurrence of *Cronobacter* spp. and *Cronobacter sakazakii* in US retail foods

For comparison purposes, a survey of US retail foods was conducted for the period of March 2022 to August 2023. Food items which demonstrated significant (~10% or greater) *Cronobacter* spp. contamination frequencies included whole grain/baked goods/flours (26.3%), sprouted seeds/seeds/beans (15.3%), nuts/nut butters (14.2%), and candies/snacks/ desserts (9.3%) (Table 2). *C. sakazakii* contamination frequencies of food items showed a similar pattern to that of *Cronobacter* spp. The highest frequency of contamination was observed for whole grain/baked goods/flours (25.8%). Of the 4,009 food samples tested, 6.0% were positive for *Cronobacter* spp. and 5.1% were positive for *C. sakazakii,* with residual isolates (0.0%–0.3% of total isolates) identified as *C. turicensis, C. dublinensis*, *C. malonaticus, C. muytjensii,* and *C. universalis* (in descending order of occurrence).

**TABLE 2:** Recovery of *Cronobacter* spp. from US retail foods

Retail food categories^*a*^	No. of samples tested	*Cronobacter* spp. positives no. (%)	*C. sakazakii* positives no. (%)	*C. turicensis* positives no. (%)	*C. malonaticus* positives no. (%)	*C. dublinensis* positives no. (%)	*C. muytjensii* positives no. (%)	*C. universalis* positives no. (%)
Grains/baked goods/flours	414	109 (26.3)	107 (25.8)	1 (0.2)	0 (0)	1 (0.2)	0 (0)	0 (0)
Seeds/sprouts/ beans^*b*^	222	34 (15.3)	22 (9.9)	1 (0.5)	1 (0.5)	7 (3.2)	2 (0.9)	1 (0.5)
Nuts/nut butter	288	41 (14.2)	29 (10.1)	5 (1.7)	1 (0.3)	4 (1.4)	1 (0.3)	1 (0.3)
Candy/dessert/snack^*c*^	54	5 (9.3)	5 (9.3)	0 (0)	0 (0)	0 (0)	0 (0)	0 (0)
Botanical/nutraceutical/supplement	241	12 (5.0)	12 (5.0)	0 (0)	0 (0)	0 (0)	0 (0)	0 (0)
Pet food	439	17 (3.9)	15 (3.4)	0 (0)	2 (0.5)	0 (0)	0 (0)	0 (0)
Spice/seasoning	68	2 (2.9)	1 (1.5)	1 (1.5)	0 (0)	0 (0)	0 (0)	0 (0)
Trail mix/snack mix	1,679	19 (1.1)	13 (0.8)	2 (0.1)	4 (0.2)	0 (0)	0 (0)	0 (0)
Fruits/vegetables	169	0 (0)	0 (0)	0 (0)	0 (0)	0 (0)	0 (0)	0 (0)
Noodles/pasta	94	0 (0)	0 (0)	0 (0)	0 (0)	0 (0)	0 (0)	0 (0)
Beef^*d*^	299	0 (0)	0 (0)	0 (0)	0 (0)	0 (0)	0 (0)	0 (0)
Chicken^*e*^	42	0 (0)	0 (0)	0 (0)	0 (0)	0 (0)	0 (0)	0 (0)
Total	4,009	239 (6.0)	204 (5.1)	10 (0,2)	8 (0.2)	12 (0.3)	3 (0,1)	2 (0.0)

^*a*^ Culture-confirmed positive samples are expressed as the number of positive sites and parenthetically as the percentage of total samples in each category.

^*b*^ Samples consisting mostly of sesame, chia, buckwheat, wheat berries, alfalfa, sunflowers, and various beans.

^*c*^ Samples consisting mostly of milk or dark chocolate candies, mints, chocolate-coated berries, and chocolate- or yogurt-coated pretzels.

^*d*^ Samples consisting mostly of ground beef (various fat content), chuck roast, sirloin, and heart.

^*e*^ Samples consisting mostly of ground chicken, chicken wings, and chicken-based sausage.

### Genetic composition and diversity *of Cronobacter* spp. isolates

**Fig 1:**
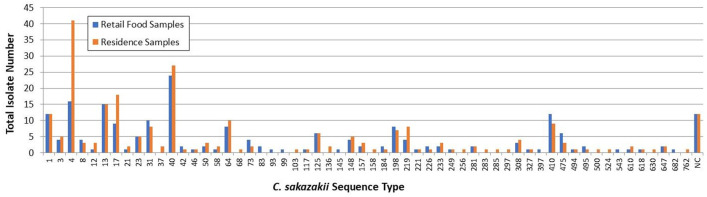
Distribution of *Cronobacter sakazakii* MLST obtained from retail foods (blue) and US residences (orange).

To ascertain a possible relationship between *C. sakazakii* isolates obtained from the domestic environmental samples and isolates recovered from retail foods, seven-locus multi-locus sequence typing was performed (Fig. 1) using the whole genomic sequences. For US homes (*N* = 248 *C*. *sakazakii* isolates total), 12 isolates (5%) did not match to previously assigned STs, and the remaining 236 isolates (95%) conformed to 51 different sequence types. The sequence type most commonly isolated from US homes was ST4, followed by ST40, ST17, ST13, ST1, and ST64. Correspondingly, for retail foods (*N* = 203 *C*. *sakazakii* isolates total), 12 isolates (6%) did not match to a previously defined ST, and the remaining 192 isolates (94%) matched to 45 different STs, with ST40 identified as the most common sequence type, followed by ST4, ST13, ST1, ST410, and ST1.

In comparison, the most commonly documented *C. sakazakii* ST at the MLST database (https://pubmlst.org/bigsdb?db=pubmlst_cronobacter_isolates, 2,322 records) is ST4, followed by ST1, ST8, ST64, ST13, and ST40 (data not shown). Although not entirely overlapping, four of the top six sequence types are common to samples collected from homes and retail foods as well as the MLST database. Importantly, the most clinically significant *C. sakazakii* STs reported in the literature are ST1, ST4, ST8, and ST12 (48), where ST4 is the pathovar most commonly associated with meningitis in neonates (12, 13). Aside from ST4, no other STs belonging to clonal complex 4 were identified in this survey.

**Fig 2:**
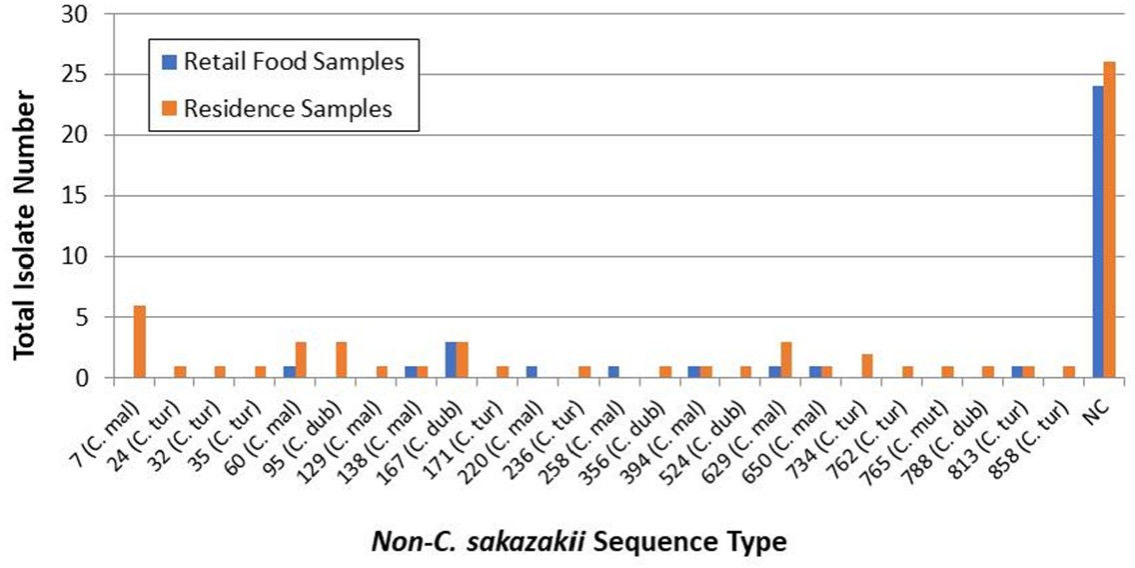
Distribution of non-*Cronobacter sakazakii* MLST obtained from retail foods (blue) and US residences (orange).

Analysis of non-*sakazakii* species isolated from foods and domestic environmental samples (*N* = 97 isolates total) identified 50 isolates (52%) which were unique and did not match to known STs with the remaining 47 isolates (48%) matching to 24 established STs (Fig. 2). The most frequent non-*sakazakii* ST isolated from retail foods was ST167 *C. dublinensis* (three isolates) and, from homes, ST7 *C. malonaticus* (six isolates). Of note, ST7 is the most common sequence type isolated from adults with *Cronobacter* infections (49).

WGS comparisons made using 386 high-quality sequences obtained from isolates of both foods and homes revealed significant genetic diversity. With the exception of isolates collected from the same home, the remaining genomes (*N* = 269) were entirely unique and 118 clustered into 47 groups. None of the sequences were identical (SNPs <5) to sequences previously deposited at the NCBI pathogen browser. Several similarities were found with previously banked sequences. An isolate collected from a US home showed similarity (differing by 29 SNPs) to an isolate from an environmental sponge collected from a food manufacturing plant roughly 6 months apart in a separate state. This isolate was identified as ST12, a *C. sakazakii* pathovar associated with NEC in infants (50). A second isolate from a US home clustered with a 2008 food isolate, differing by 43 SNPs. Lastly, a third isolate obtained from a US home identified as ST4 clustered with a number of clinical isolates collected from 2003 to 2016 as well as environmental samples, including isolates from PIF (2011), breast milk (2016), and a breast pump (2011). In this instance, the genomic sequence differed by 50 or more SNPs.

**Fig 3:**
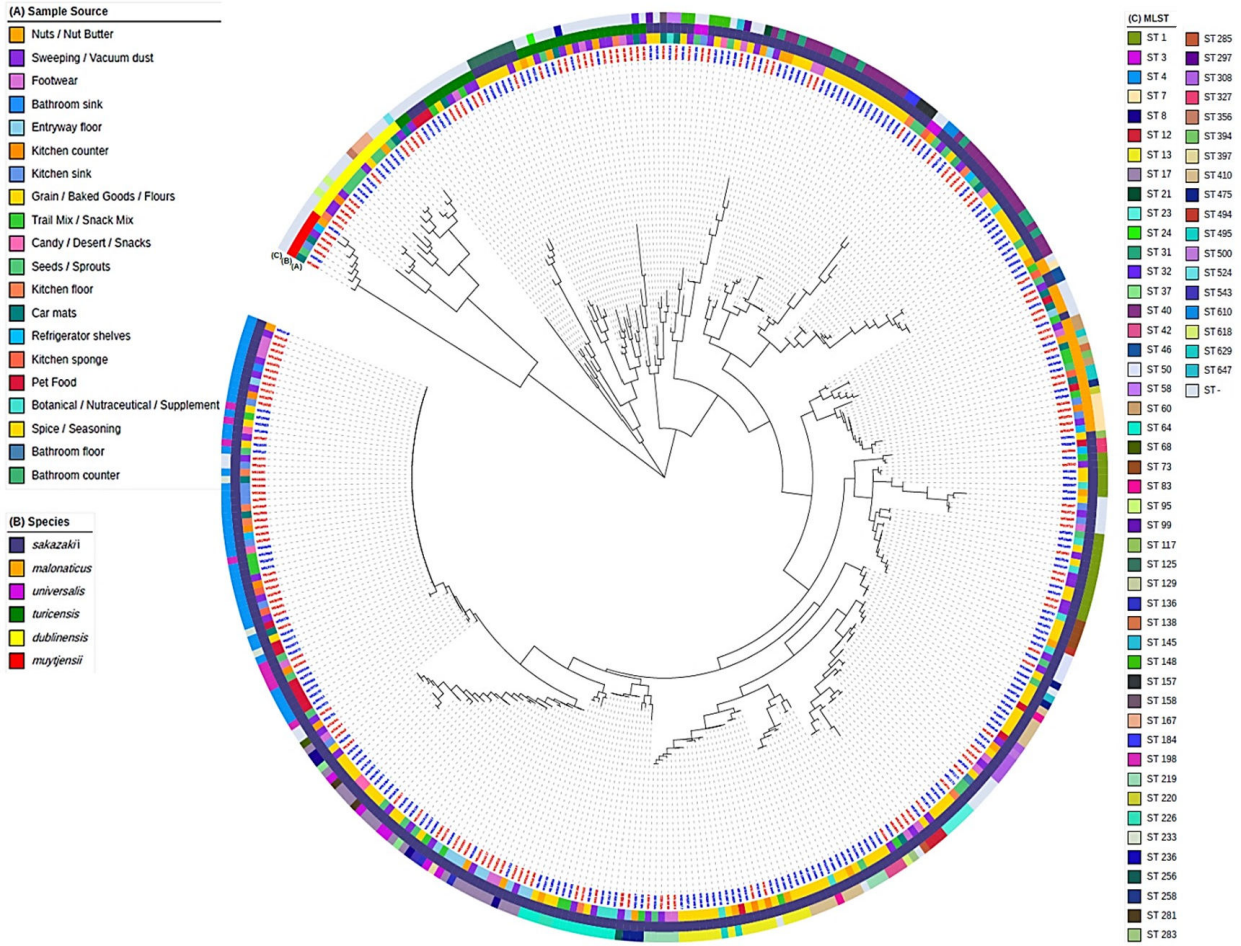
Phylogenetic analysis of *Cronobacter* spp. isolates based on MLST allele profiles. WGS values indicated by blue lettering are isolates obtained from retail foods, and WGS values indicated by red lettering are isolates derived from US residences. NC refers to isolates that did not conform to previously defined sequence types.

Genetic diversity was further analyzed by assessing the population structure of 386 high-quality *Cronobacter* spp. genomic sequences based on assessment of MLST loci sequences (Fig. 3). While isolates clustered by ST, they did not robustly cluster by sample class (either retail foods or residences) nor by specific categories or foods or domestic isolation sites. Of note, sizable recovery of *C. sakazakii* ST4 was observed for the pet food samples (1.4%, 6 isolated from 439 pet food samples), implying a novel transmission vehicle in the domestic environment. Tree nuts and peanuts also showed notable recovery of *C. sakazakii* ST4 (1.0%, 3 isolates out of 288 samples). Within the domestic setting, ST4 was most commonly recovered from kitchen surfaces (17 isolates), followed by floors (not including kitchen floors, 11 isolates).

### Virulence features and antibiotic resistance markers of *Cronobacter* spp. isolates

Potential virulence and clinical significance were assessed in 386 sequenced isolates using the web-based bioinformatics tool BV-BRC (45). Included in the analysis were markers of secretion system components, host invasion/resistance factors, toxins, adhesion factors, invasion and host resistance factors, and antibiotic resistance enzymes/efflux pumps. The occurrence of each marker in the 386 genomes is indicated in Table 3; markers that were not represented in the sequences were excluded in the tabulation. Of the six different secretion systems, T1SS, T3SS, and T6SS were identified in most or all of the genomes. Evidence of T2SS was not identified in any of the genomes. T1SS was absent in some *C. dublinensis* isolates. T4SS, identified in only 27 genomes, was found primarily in a subset of *C. sakazakii* isolates, with no ST preference, and a few other species. A putative enterotoxin with similarity to EspC from enteropathogenic *E. coli* was identified in 24 different genomes, including *C. sakazakii and C. malonaticus*, with four isolates designated as ST4 but none as ST7 or ST12. Adhesion factors associated with virulence were variably represented. Curli (represented by *CsgC*), which facilitates adhesion and invasion into host cells was identified in 40 genomes, most of which were *C. malonaticus,* with no ST bias observed. P pilus (represented by PapA) and type 1 pili (represented by FimA) facilitate colonization of the upper urinary tract by uropathogenic *E. coli*. These were documented in 301 and 285 genomes, respectively, while their absence was noted primarily for *C. sakazakii* isolates.

**TABLE 3:** Frequency of putative virulence factors and antibiotic resistance factors present in 386 *Cronobacter* spp. isolates

Class	Product	Marker/gene	Putative function	Number of genomes containing
Secretion system	T1SS	HlyD	Secrete adhesins, iron-scavenging proteins, lipases, proteases, pore-forming toxins	378
	T3SS	Flha	Secrete flagellar and other components	386
	T4SS	VirB8	Secrete/take up macromolecules, inject virulence factors into host cells	27
	T5SS	Fha	Promotes surface attachment, tissue colonization, and linked to biofilm formation	363
	T6SS	TssB	Primarily involved in outcompeting with other bacteria	386
Adhesion	Curli	CsgC	Adhesion to surfaces, cell aggregation, and biofilm formation; adhesion and invasion of host cells	40
	P pilus	PapA	Colonization of the kidney	301
	Sigma-fimbriae	Sigma usher protein	Biofilm formation	386
	Type I pilus	FimA	Adherence to mucosal surfaces and inflammatory cells *in vitro*	285
	Type IV pilus	PilA	Locomotion, adherence to host cells, DNA uptake, and protein secretion	386
Toxins	EspC	EPEC-secreted protein C	Enterotoxin	24
Invasion and resistance factors	*Cronobacter* plasminogen activator (Omptin)	Cpa	Protease, enables persistence in blood by activating plasminogen and inactivating α2-antiplasmin and complement components	319
	Cronobactin	EfeO	Iron acquisition, contributes to the systemic survival, dissemination and invasion of the CNS	386
	Capsule	SCO6023	Exopolysaccharide phosphotransferase, adherence to surfaces (biotic/abiotic), biofilm formation, evasion from opsonization	386
	Enterobactin	Ferric enterobactin transporter	Iron acquisition	386
	Macrophage infectivity potentiator	FkpA (Mip)	FKBP-type peptidyl-prolyl *cis-trans* isomerase, enables survival and persistence within macrophage	386
	RNA chaperone	Hfq	Translational regulator involved in invasion of cells, dissemination, and stress adaptation	386
	Type III hemolysin	HlyIII	Enables persistence in blood through beta-hemolytic or α-hemolytic activity	386
	IbeB efflux system	CusC, SilC	Copper and silver resistance cation efflux system, facilitates invasion of brain microvascular endothelial cells	125
	Invasin, putative	Inv	Contributes to invasion of organs	386
	Siderophore-mediated iron acquisition IucABCD	IucA	Iron acquisition, contributes to the systemic survival and dissemination as well as subsequent invasion of the CNS	384
	Sialic acid utilization system NanAKT	NanR	Enables utilization of sialic acid which is found in breast milk, infant formula, intestinal mucin, and gangliosides in the brain; enables adaptation to the milk powder environment as well as invasion in the intestine and the CNS	332
	Outer membrane protein A	OmpA	Involved in invasion of enterocyte-like epithelial cells and endothelial cells; contributes to invasion of CNS through interaction with brain microvascular endothelial cells	386
	Outer membrane protein X	OmpX	Involved in invasion of enterocyte-like epithelial cells, endothelial cells, and organs	386
	Two-component regulatory system PhoP/PhoQ	PhoP	Regulation of lipid A modification, regulation of resistance to antimicrobial peptides	386
	Two-component regulatory system PmrA/PmrB	PmrA	Resistance to antimicrobial peptides	386
	Lipid A phosphoethan-olamine transferase	PmrC	Resistance to antimicrobial peptides	386
	Superoxide dismutase	SodA	Superoxide dismutase that promotes survival in macrophages by opposing oxidative stress	386
	Colanic capsule	WcaD	Colanic capsule protects from desiccation and aids in biofilm formation	386
Antibiotic resistance	Aminoglycoside 3*''*-phosphotransferase	Aph	Resistance to aminoglycosides	1
	Beta lactamase, subclass B3	Beta lactamase, subclass B3	Resistance to carbapenems	354
	Beta lactamase, class C	Beta lactamase, class C	Resistance to cephalosporins	386
	Fosfomycin-modifying metalloglutathione	FosA	Resistance to fosfomycin	386
Efflux pumps involved in antibiotic resistance	AcrAD-TolC	AcrD	Multidrug resistance	386
	AcrEF-TolC	AcrE	Multidrug resistance	366
	EmrAB-OMF	EmrA	Multidrug resistance	386
	MacAB-TolC	MacA	Multidrug resistance	386
	Multiple antibiotic resistance MarABR	MarA	Upregulates efflux pumps involved in resistance to multiple antibiotics	386
	MdfA	MdfA	Resistance to tetracyclines	386
	MdtABC-TolC	MdtD	Resistance to aminocoumarins	386
	MsbAB-TolC	MsbA	Multidrug resistance	386
	Tetracycline efflux pump TetAGW (TetA)	TetA	Resistance to tetracyclines	2
	YkkCD	YkkC	Resistance to tetracyclines, phenicols, aminoglycosides	1

Analysis of antibiotic resistance markers indicated that most or all genomes contained genes for antibiotic resistance enzymes implicated in resistance to carbapenems and cephalosporins. Likewise, all genomes contained genes coding for efflux pumps with broad *in silico* predicted specificity for aminoglycosides, fluoroquinolones, macrolides, tetracyclines, aminocoumarins, and nitroimidazoles. With respect to factors involved in infectivity and host resistance, several markers were found to occur with high frequency across the 386 genomes. The gene coding for Cpa (Omptin), which confers resistance to serum, was found in 319 genomes. Macrophage infectivity potentiator (*MIP*), superoxide dismutase (*SOD*), and type III hemolysin (*HlyIII*) were found in all 386 genomes. Other putative virulence factors associated with *Cronobacter* infections included *OmpA* (all 386 genomes), *OmpX* (386 genomes), *IbeB* (125 genomes), *IucABCD* (384 genomes), and the *NanAKT* cluster (332 genomes). Of note, evidence of *IbeB* and *NanAKT* clusters was seen in most but not all *C. sakazakii* isolates. These two clusters were also observed in the genomes of a few other species.

To gain further insight into the significance of the virulence factors, the analysis was further refined by examining the genetic variation of key virulence factors present in the five most common *Cronobacter sakazakii* STs identified by this survey: ST1 (*N* = 18), ST4 (*N* = 44), ST13 (*N* = 16), ST17 (*N* = 20), and ST40 (*N* = 33), representing a total of 131 genomes (or 33.9% of the total *N* = 386 genomes). Of note, ST1 and ST4 are clinically relevant strains, whereas ST13, ST17, and ST40 are not. As indicated in Table 4 (boldface), a number of alleles segregated with pathovar sequence types. In this regard, all ST4 isolates shared unique alleles for *SOD*, *HlyIII*, *EfeO*, *IucA*, and *Cpa*.

**TABLE 4:** Nested analysis of putative virulence factors in *Cronobacter sakazakii* ST1, ST4, ST13, ST17, and ST40 isolates

Virulence factor	Pathovar ST		Non-pathovar ST		
	ST1 inferred alleles^*a*^	ST4 inferred alleles	ST13 inferred alleles	ST17 inferred alleles	ST40 inferred alleles
	*N* = 18	*N* = 44	*N* = 16	*N* = 20	*N* = 33
*Cpa*	**0(1), 1**(**17**)	2(44), 3(1)*	4(7), 5(9)	6(19), 7(1)	7(33)
*EfeO*	**1(16), 2**(**2**)	**3(38) ,4**(**6**)	5(16)	6(19), 7(1)	8(33)
*Fha*	**1(13), 2**(**5**)	**2(42), 3**(**2**)	4(9), 5(7)	6(19), 7(1)	8(9), 9(22), 10(1), 11(1)
*FkpA (Mip*)	1(18)	**2(3), 3**(**41**)	1(16)	4(20)	5(33)
*Hfq*	1(18)	1(44)	1(16)	1(20)	2(31), 3(2)
*Hha*	1(18)	1(44)	2(16)	1(20)	2(33)
*HlyIII*	**1**(**18**)	2(44), 3(1)*	4(14), 5(2)	6(20)	7(33)
*IbeB (CusC, SilC*)	0(1), 1(16), 2(2)*	0(9), **2(10),** 3(20), 4(2)*, 5(2)*, 6(1)*, 7(1)*, 8(1), 9(1)	0(13), 9(2), 10(1)	0(19), 11(1)	0(24), 3(1), 10(8)
*IucABCD/ IutA*	**0(1), 1(16), 2**(**1**)	**3 (28), 4 (1), 5 (15**)	6(7), 7(9)	8(20)	9(33)
*NanAKT (NanR*)	1(18)	**2(42),** 3(2)	1(16)	3(19), 4(1)	4(33)
*SodA*	**1(16),** 2(2)	**3**(**44**)	2(16)	4(20)	2(33)
*WcaD*	**1**(**18**)	**2(9), 3(33),** 4(2)	5(15), 6(1)	4(20)	4(33)

^*a*^ Inferred alleles indicated by boldface type segregate with pathovars ST1 and/or ST4. *N* = number of genomes for that sequence type. Numbers in parentheses refer to the number of occurrences of an inferred allele for a given sequence type. Alleles marked with an asterisk (*) indicate duplication of a gene within one or more of the genomes.

## DISCUSSION

*Cronobacter* spp. exhibit a wide-ranging ecological distribution, a property that can be attributed to their ability to withstand adverse conditions such as acid stress, temperature stress, prolonged desiccation, exposure to disinfectants, and osmotic stress (51–57). Recovery of *Cronobacter* spp. has been documented for a wide variety of environments and foods (55–61). With respect to foods, *Cronobacter* spp. have been most frequently recovered from plant-based foods, such as vegetables, legumes, fruits, sprouts, cereal products, flour-based confections, herbs, nuts, teas, and spices (29, 30, 58–71). This current survey of US retail foods similarly identified substantial contamination of plant-based products. The most frequently contaminated categories included grains/baked goods/flours (26.3% of samples were positive). However, recoveries from spices/seasonings, fruits, raw meat, and vegetables were negligible. This discrepancy may reflect distinctions in HACCP practices in different countries, such as the use of irradiation in the US or changes in quality control standards over time. Indeed, with the exception of one study examining foods from 44 countries (70), the remaining food studies have characterized *Cronobacter* burden in retail foods from outside North America, including from China, Korea, the Czech Republic, Slovakia, Jordan, India, Brazil, Ireland, and Switzerland. An interesting outcome of the retail food analysis was that the ST distribution profile of *C. sakazakii* isolates essentially mirrored that of isolates obtained from US homes, with biased frequencies noted for ST1, ST4, ST13, ST17, and ST40 in both sample categories. The occurrence of pathovars ST1 and ST4 is significant (48). The significance of the prevalence of ST4 is most notable due to its association with neonatal meningitis (13). Indeed, these distributions were similar to the ST distribution at the MLST database. These similarities may simply reflect the natural ecological distribution of different sequence types*,* though it is tempting to speculate that this apparent similarity may reflect a causal relationship between retail foods and introduction of organisms into the home setting. Similarly, the prevalence of *Cronobacter* spp. isolated from entry hallways and floors suggests that foot traffic may also serve as a vehicle for entry into the domestic environment as well.

Although not addressed by this study, *Cronobacter* spp. have also been isolated from powdered infant formulas, infant cereals, and powdered milk (54, 61, 72, 73). Indeed, PIF is recognized around the world as a primary vehicle for transmission to infants (10, 18–22, 74). The connection with PIF can be attributed to several clustered cases and hospital-associated outbreaks that occurred in the Netherlands (1977–1981) (74), Tennessee, USA (1988) (18), France (1994) (22), Belgium (1998) (19), and the US across multiple states (2011) (13). However, a patient-linked organism was only recovered from unopened cans of PIF in the Belgium outbreak; in the remainder, recovery of patient-linked organism was only made from previously opened or previously prepared PIF (19). PIF, which is not a sterile product, is inherently prone to microbiological outgrowth if not handled according to the manufacturer’s guidelines (21). Several outbreaks occurring in neonatal intensive care units have been attributed to inadequate hygienic preparation and temperature control of reconstituted PIF (20, 22). The earliest microbiological survey of PIF published in 1988 indicated that 14% (20 of 141) of samples collected from 35 countries were contaminated with *Cronobacter* spp. (*Enterobacter sakazakii*) (17). A field survey conducted by the FDA in 2003 found *Cronobacter* spp. in 22.7% (5 of 22) finished product samples (unpublished findings). A more recent 2017 survey conducted in China, which assessed over 6,000 infant formula samples, reported a *C. sakazakii* contamination rate of <0.5% (73).

Of note, US source investigations into cases of invasive *Cronobacter* infection in infants have been unable to link infections to unopened (intrinsically) contaminated PIF containers (10). Indeed, the only reported instance in the US of linkage with any type of powdered formula occurred in a Tennessee neonatal intensive care unit (NICU) in 2001. This case linked a clinical isolate to unopened containers of Portagen, a formula type intended for children and adults but not infants and therefore not subject to the same microbiological testing criteria and GMP requirements that were implemented by the FDA in 2014 (20). This case was the first and last instance of intrinsically contaminated powdered formula linked to infant infections in the US, though notably, it did not involve PIF. Accordingly, it remains highly plausible that the contamination events causing US infections are occurring in the hospital or community setting. Supporting the role for the environment in the transmission to infants is the fact that most NICUs in the US no longer use PIF due to a 2002 recommendation by the FDA to avoid feeding it in neonatology units. Yet since 2004, hospital-acquired infections have represented 23%–33% of invasive illness cases in infants [(10), unpublished review of CDC cases 2020–2023]. Furthermore, clustered, community-onset cases have never been epidemiologically linked to one another, implying a unique origin in each instance.

A number of studies have been undertaken to determine the occurrence of *Cronobacter* spp. in the domestic environment. Source investigation findings have linked 44% (31 of 71) of cases involving invasive illness in infants with environmental samples collected from homes. Furthermore, surveillance studies have documented *Cronobacter* spp. contamination in 31% (5 of 16 homes) of Dutch households (32), *C. sakazakii* contamination in 24.7% (21 of 85 homes) of Czech households (29), and *C. sakazakii* contamination in 26.9% (21 of 78 homes) of Tennessee households (28). A 2016 surveillance study of domestic households in Georgia showed that 78.5% (51 of 65) of homes tested positive for *Cronobacter* spp. (27). This outcome is considerably higher than the outcomes obtained in the current study or abovementioned studies, and may reflect methodological differences. Within the domestic environment, *Cronobacter* spp. have been reproducibly isolated from vacuum cleaner contents, threshold floors, refrigerators, sinks, kitchen floors, cleaning cloths, and kitchen sponges (27–33), essentially mirroring sites of higher contamination identified by the current study.

Assessment of pathogenic potential indicated that many of the isolates shared genetic features that facilitate hematogenous spread as well as virulence and pathogenicity by uropathogenic and enteropathogenic bacteria. These findings help explain the ability of *Cronobacter* to cause opportunistic infections of the blood, urinary, and gastro-intestinal systems. Similarly, the genomes contained a high proportion of antibiotic resistance genes that could potentially enable the organisms to evade an array of different antimicrobial and antiseptic classes. The identification of 14 antibiotic resistance marker genes, including those that rely on target alteration and efflux systems, is similar to the 19 that have been previously reported for 5 *Cronobacter* spp. isolates (75). A key distinction in this study was the occurrence of a marker for resistance to aminoglycosides in one of the isolates. While the identification of these factors at the genetic level does not guarantee their functionality, the widespread occurrence of many of these factors suggests that the *Cronobacter* spp. found in homes and on foods pose certain potential to cause disease. Furthermore, nested assessment of genetic variation in putative virulence factors revealed a pattern of inferred alleles, where alleles for *SOD*, *HlyIII*, *EfeO*, *IucA*, and *Cpa* appeared to segregate with pathovars ST1 and ST4, as opposed to ST13, ST17, and ST40. Of note, *HlyIII* is thought to enable persistence in blood through α- or beta-hemolytic activity. *EfeO* is associated with persistence and dissemination in the blood, as well as invasion of the CNS. *Cpa* enables persistence in blood through inactivation of α2-antiplasmin and complement components. In the absence of functional studies, this segregation of inferred VF alleles does not necessarily denote virulence causality. An alternate explanation for the apparent VF allelic segregation may simply involve clonality and, accordingly, warrants further detailed analyses.

This study contributes additional understanding obtained through detailed genetic analysis. In particular, the profound genetic diversity identified by this study helps explain the sporadic, epidemiologically unlinked nature of infections in the US. Furthermore, the dominance of pathovars ST1 and ST4 in retail foods and domestic samples provides insight into a potential exposure route to clinically significant strains in the home.

Additionally, these findings extend our understanding of *Cronobacter* transmission dynamics via the following inferences. First, introduction of *Cronobacter* into the home appears to relate to foot traffic, as supported by the contamination rates of entryway floors, footwear, and sweeping/vacuum cleaner dust reported in our study (Table 1). This risk could be mitigated by restricting use of outdoor footwear within the home and enhancing floor cleaning measures such as the use of approved disinfectants and vacuum cleaners fitted with HEPA filters. Second, the kitchen poses significant risk for food contamination, and the adoption of improved hygiene measures in the kitchen, including the use of approved disinfectants, may mitigate this risk. Third, the possibility that certain retail foods, including pet food, may be introducing *Cronobacter* into the home, implying the need to handle high-risk foods with more caution. Fourth, these data suggest that fecal-oral transmission is likely insignificant based on the paucity of isolates recovered from bathroom surfaces. Importantly, while these findings do not preclude the need to maintain stringent manufacturing practices and rigorous product testing, they do support the need to improve hygienic measures in the home setting.

## SUPPLEMENTAL MATERIAL

Supplemental material aem.00700-24-s0001.docx NCBI BioSample ID list.

ASM does not own the copyrights to Supplemental Material that may be linked to, or accessed through, an article. The authors have granted ASM a non-exclusive, world-wide license to publish the Supplemental Material files. Please contact the corresponding author directly for reuse.

## References

[1] Farmer JJ, Asbury MA, Hickman FW, Brenner DJ, The Enterobacteriaceae Study Group. 1980. Enterobacter sakazakii: a new species of “Enterobacteriaceae” isolated from clinical specimens. Int J Syst Bacteriol 30:569–584. doi:10.1099/00207713-30-3-569

[2] Iversen C, Lehner A, Mullane N, Bidlas E, Cleenwerck I, Marugg J, Fanning S, Stephan R, Joosten H. 2007. The taxonomy of Enterobacter sakazakii: proposal of a new genus Cronobacter gen. nov. and descriptions of Cronobacter sakazakii comb. nov. Cronobacter sakazakii subsp. sakazakii, comb. nov., Cronobacter sakazakii subsp. malonaticus subsp. nov., Cronobacter turicensis sp. nov., Cronobacter muytjensii sp. nov., Cronobacter dublinensis sp. nov. and Cronobacter genomospecies 1. BMC Evol Biol 7:64. doi:10.1186/1471-2148-7-64 17439656 PMC1868726

[3] Iversen C, Mullane N, McCardell B, Tall BD, Lehner A, Fanning S, Stephan R, Joosten H. 2008. Cronobacter gen. nov., a new genus to accommodate the biogroups of Enterobacter sakazakii, and proposal of Cronobacter sakazakii gen. nov., comb. nov., Cronobacter malonaticus sp. nov., Cronobacter turicensis sp. nov., Cronobacter muytjensii sp. nov., Cronobacter dublinensis sp. nov., Cronobacter genomospecies 1, and of three subspecies, Cronobacter dublinensis subsp. dublinensis subsp. nov., Cronobacter dublinensis subsp. lausannensis subsp. nov. and Cronobacter dublinensis subsp. lactaridi subsp. nov. Int J Syst Evol Microbiol 58:1442–1447. doi:10.1099/ijs.0.65577-0 18523192

[4] Joseph S, Desai P, Ji Y, Cummings CA, Shih R, Degoricija L, Rico A, Brzoska P, Hamby SE, Masood N, Hariri S, Sonbol H, Chuzhanova N, McClelland M, Furtado MR, Forsythe SJ. 2012. Comparative analysis of genome sequences covering the seven Cronobacter species. PLoS ONE 7:e49455. doi:10.1371/journal.pone.0049455 23166675 PMC3500316

[5] Healy B, Cooney S, O’Brien S, Iversen C, Whyte P, Nally J, Callanan JJ, Fanning S. 2010. Cronobacter (Enterobacter sakazakii): an opportunistic foodborne pathogen. Foodborne Pathog Dis 7:339–350. doi:10.1089/fpd.2009.0379 19958103

[6] Holý O, Forsythe S. 2014. Cronobacter spp. as emerging causes of healthcare-associated infection. J Hosp Infect 86:169–177. doi:10.1016/j.jhin.2013.09.011 24332367

[7] Holý O, Petrželová J, Hanulík V, Chromá M, Matoušková I, Forsythe SJ. 2014. Epidemiology of Cronobacter spp. isolates from patients admitted to the Olomouc university hospital (Czech Republic). Epidemiol Mikrobiol Imunol 63:69–72. 24730997

[8] Alsonosi A, Hariri S, Kajsík M, Oriešková M, Hanulík V, Röderová M, Petrželová J, Kollárová H, Drahovská H, Forsythe S, Holý O. 2015. The speciation and genotyping of Cronobacter isolates from hospitalised patients. Eur J Clin Microbiol Infect Dis 34:1979–1988. doi:10.1007/s10096-015-2440-8 26173692 PMC4565866

[9] Jason J. 2015. The roles of epidemiologists, laboratorians, and public health agencies in preventing invasive Cronobacter infection. Front Pediatr 3:110. doi:10.3389/fped.2015.00110 26734593 PMC4689785

[10] Strysko J, Cope JR, Martin H, Tarr C, Hise K, Collier S, Bowen A. 2020. Food safety and invasive Cronobacter infections during early infancy, 1961-2018. Emerg Infect Dis 26:857–865. doi:10.3201/eid2605.190858 32310746 PMC7181934

[11] Patrick ME, Mahon BE, Greene SA, Rounds J, Cronquist A, Wymore K, Boothe E, Lathrop S, Palmer A, Bowen A. 2014. Incidence of Cronobacter spp. infections, United States, 2003–2009. Emerg Infect Dis 20:1520–1523. doi:10.3201/eid2009.140545 25148394 PMC4178417

[12] Joseph S, Forsythe SJ. 2011. Predominance of Cronobacter sakazakii sequence type 4 in neonatal infections. Emerg Infect Dis 17:1713–1715. doi:10.3201/eid1709.110260 21888801 PMC3322087

[13] Hariri S, Joseph S, Forsythe SJ. 2013. Cronobacter sakazakii ST4 strains and neonatal meningitis, United States. Emerg Infect Dis 19:175–177. doi:10.3201/eid1901.120649 23260316 PMC3557988

[14] Kucerova E, Clifton SW, Xia X-Q, Long F, Porwollik S, Fulton L, Fronick C, Minx P, Kyung K, Warren W, Fulton R, Feng D, Wollam A, Shah N, Bhonagiri V, Nash WE, Hallsworth-Pepin K, Wilson RK, McClelland M, Forsythe SJ. 2010. Genome sequence of Cronobacter sakazakii BAA-894 and comparative genomic hybridization analysis with other Cronobacter species. PLoS ONE 5:e9556. doi:10.1371/journal.pone.0009556 20221447 PMC2833190

[15] Lai KK. 2001. Enterobacter sakazakii infections among neonates, infants, children, and adults. Case reports and a review of the literature. Medicine (Baltimore) 80:113–122. doi:10.1097/00005792-200103000-00004 11307587

[16] Minor T, Lasher A, Klontz K, Brown B, Nardinelli C, Zorn D. 2015. The per case and total annual costs of foodborne illness in the United States. Risk Analysis 35:1125–1139. doi:10.1111/risa.12316 25557397

[17] Muytjens HL, Roelofs-Willemse H, Jaspar GH. 1988. Quality of powdered substitutes for breast milk with regard to members of the family Enterobacteriaceae. J Clin Microbiol 26:743–746. doi:10.1128/jcm.26.4.743-746.1988 3284901 PMC266435

[18] Simmons BP, Gelfand MS, Haas M, Metts L, Ferguson J. 1989. Enterobacter sakazakii infections in neonates associated with intrinsic contamination of a powdered infant formula. Infect Control Hosp Epidemiol 10:398–401. doi:10.2307/30144207 2794464

[19] van Acker J, de Smet F, Muyldermans G, Bougatef A, Naessens A, Lauwers S. 2001. Outbreak of necrotizing enterocolitis associated with Enterobacter sakazakii in powdered milk formula. J Clin Microbiol 39:293–297. doi:10.1128/JCM.39.1.293-297.2001 11136786 PMC87717

[20] Himelright I, Harris E, Lorch V, Anderson M, Jones T, Craig A, Kuehnert M, Forster T, Arduino M, Jensen B, Jernigan D. 2002. Enterobacter sakazakii infections associated with the use of powdered infant formula --- Tennessee, 2001. MMWR 51:298-300. Available from: https://www.cdc.gov/mmwr/preview/mmwrhtml/mm5114a1.htm. Retrieved 4 Aug 2024.

[21] FAO/WHO. 2004. Enterobacter sakazakii and other microorganisms in powdered infant formula: meeting report, 2004. Available from: https://www.who.int/publications/i/item/9789241562775. Retrieved 4 Aug 2024.

[22] Caubilla-Barron J, Hurrell E, Townsend S, Cheetham P, Loc-Carrillo C, Fayet O, Prère M-F, Forsythe SJ. 2007. Genotypic and phenotypic analysis of Enterobacter sakazakii strains from an outbreak resulting in fatalities in a neonatal intensive care unit in France. J Clin Microbiol 45:3979–3985. doi:10.1128/JCM.01075-07 17928419 PMC2168550

[23] Baumbach J, Rooney K, Smelser C, Torres P, Bowen A, Nichols M. 2009. Cronobacter species isolation in two infants --- New Mexico, 2008. MMWR 58:1179-1183. Available from: https://www.cdc.gov/mmwr/preview/mmwrhtml/mm5842a3.htm. Retrieved 4 Aug 2024.

[24] Haston JC, Miko S, Cope JR, McKeel H, Walters C, Joseph LA, Griswold T, Katz LS, Andújar AA, Tourdot L, Rounds J, Vagnone P, Medus C, Harris J, Geist R, Neises D, Wiggington A, Smith T, Im MS, Wheeler C, Smith P, Carleton HA, Lee CC. 2023. Cronobacter sakazakii infections in two infants linked to powdered infant formula and breast pump equipment — United States, 2021 and 2022. MMWR Morb Mortal Wkly Rep 72:223–226. doi:10.15585/mmwr.mm7209a2 36862586 PMC9997662

[25] Bowen A, Wiesenfeld HC, Kloesz JL, Pasculle AW, Nowalk AJ, Brink L, Elliot E, Martin H, Tarr CL. 2017. Notes from the field: Cronobacter sakazakii infection associated with feeding extrinsically contaminated expressed human milk to a premature infant. MMWR Morb Mortal Wkly Rep 66:761–762. doi:10.15585/mmwr.mm6628a5 28727679 PMC5657941

[26] Sundararajan M, Enane LA, Kidwell LA, Gentry R, Danao S, Bhumbra S, Lehmann C, Teachout M, Yeadon-Fagbohun J, Krombach P, Schroeder B, Martin H, Winkjer J, Waltz T, Strysko J, Cope JR. 2018. Notes from the field: Cronobacter sakazakii meningitis in a full-term neonate fed exclusively with breast milk - Indiana, 2018. MMWR Morb Mortal Wkly Rep 67:1248–1249. doi:10.15585/mmwr.mm6744a7 30408018 PMC6223960

[27] Chan MYK. 2016. Prevalence and location of Cronobacter species and Enterobacteriacea in households. Thesis. University of Georgia. Athens Georgia. Available from: https://esploro.libs.uga.edu/esploro/outputs/graduate/Prevalence-and-location-of-Cronobacter-species/9949334442202959. Retrieved Aug 4 Aug 2024.

[28] Kilonzo-nthenge A, Rotich E, Godwin S, Nahashon S, Chen F. 2012. Prevalence and antimicrobial resistance of Cronobacter sakazakii isolated from domestic kitchens in middle Tennessee, United States. J Food Prot 75:1512–1517. doi:10.4315/0362-028X.JFP-11-442 22856579

[29] Mozrová V, Břeňová N, Mrázek J, Lukešová D, Marounek M. 2014. Surveillance and characterisation of Cronobacter spp. in Czech retail food and environmental samples. Folia Microbiol 59:63–68. doi:10.1007/s12223-013-0266-2 23873391

[5] Molloy C, Cagney C, O’Brien S, Iversen C, Fanning S, Duffy G. 2009. Surveillance and characterisation by pulsed-field gel electrophoresis of Cronobacter spp. In farming and domestic environments, food production animals and retail foods. Int J Food Microbiol 136:198–203. doi:10.1016/j.ijfoodmicro.2009.07.007 19683357

[31] Killer J, Skřivanová E, Hochel I, Marounek M. 2015. Multilocus sequence typing of Cronobacter strains isolated from retail foods and environmental samples. Foodborne Pathog Dis 12:514–521. doi:10.1089/fpd.2014.1884 25974656

[32] Kandhai MC, Reij MW, Gorris LG, Guillaume-Gentil O, van Schothorst M. 2004. Occurrence of Enterobacter sakazakii in food production environments and households. Lancet 363:39–40. doi:10.1016/S0140-6736(03)15169-0 14723994

[33] Kilonzo-Nthenge A, Chen FC, Godwin SL. 2008. Occurrence of Listeria and Enterobacteriaceae in domestic refrigerators. J Food Prot 71:608–612. doi:10.4315/0362-028X-71.3.608 18389708

[34] FDA. 2022. Available from: https://www.fda.gov/food/outbreaks-foodborne-illness/fda-investigation-cronobacter-infections-powdered-infant-formula-february-2022. Retrieved 4 Aug 2024.

[35] Newman J. 2022. Baby-formula shortage deepens, defying replenishment efforts. The Wall Street Journal. Available from: https://www.wsj.com/articles/baby-formula-shortage-deepens-defying-replenishment-efforts-11657796400. Retrieved 4 Aug 2024.

[36] FDA. BAM chapter 29: Cronobacter. Available from: https://www.fda.gov/food/laboratory-methods-food/bam-chapter-29-cronobacter. Retrieved 4 Aug 2024.

[37] Chen S, Zhou Y, Chen Y, Gu J. 2018. fastp: an ultra-fast all-in-one FASTQ preprocessor. Bioinformatics 34:i884–i890. doi:10.1093/bioinformatics/bty560 30423086 PMC6129281

[38] Andrews S. 2010. FastQC: a quality control tool for high throughput sequence data. Available from: http://www.bioinformatics.babraham.ac.uk/projects/fastqc/. Retrieved 4 Aug 2024.

[39] Gurevich A, Saveliev V, Vyahhi N, Tesler G. 2013. QUAST: quality assessment tool for genome assemblies. Bioinformatics 29:1072–1075. doi:10.1093/bioinformatics/btt086 23422339 PMC3624806

[40] Ondov BD, Treangen TJ, Melsted P, Mallonee AB, Bergman NH, Koren S, Phillippy AM. 2016. Mash: fast genome and metagenome distance estimation using MinHash. Genome Biol. 17:132. doi:10.1186/s13059-016-0997-x 27323842 PMC4915045

[41] O’Leary NA, Wright MW, Brister JR, Ciufo S, Haddad D, McVeigh R, Rajput B, Robbertse B, Smith-White B, Ako-Adjei D, et al.. 2016. Reference sequence (RefSeq) database at NCBI: current status, taxonomic expansion, and functional annotation. Nucleic Acids Res 44:D733–D745. doi:10.1093/nar/gkv1189 26553804 PMC4702849

[42] Meier-Kolthoff JP, Göker M. 2019. TYGS is an automated high-throughput platform for state-of-the-art genome-based taxonomy. Nat Commun 10:2182. doi:10.1038/s41467-019-10210-3 31097708 PMC6522516

[43] The NCBI Pathogen Detection Project. Bethesda (MD): National library of medicine (US), national center for biotechnology information. Available from: https://www.ncbi.nlm.nih.gov/pathogens/. Retrieved 4 Aug 2024.

[44] Davis S, Pettengill JB, Luo Y, Payne J, Shpuntoff A, Rand H, Strain E. 2015. CFSAN SNP pipeline: an automated method for constructing SNP matrices from next-generation sequence data. Peer J Comput Sci 1:e20. doi:10.7717/peerj-cs.20

[45] Olson RD, Assaf R, Brettin T, Conrad N, Cucinell C, Davis JJ, Dempsey DM, Dickerman A, Dietrich EM, Kenyon RW, et al.. 2023. Introducing the bacterial and viral bioinformatics resource center (BV-BRC): a resource combining PATRIC, IRD and ViPR. Nucleic Acids Res 51:D678–D689. doi:10.1093/nar/gkac1003 36350631 PMC9825582

[46] Rachlin A, Mayo M, Webb JR, Kleinecke M, Rigas V, Harrington G, Currie BJ, Kaestli M. 2020. Whole-genome sequencing of Burkholderia pseudomallei from an urban melioidosis hot spot reveals a fine-scale population structure and localised spatial clustering in the environment. Sci Rep 10:5443. doi:10.1038/s41598-020-62300-8 32214186 PMC7096523

[47] Katoh K, Misawa K, Kuma K, Miyata T. 2002. MAFFT: a novel method for rapid multiple sequence alignment based on fast fourier transform. Nucleic Acids Res 30:3059–3066. doi:10.1093/nar/gkf436 12136088 PMC135756

[48] Ogrodzki P, Forsythe SJ. 2017. DNA-sequence based typing of the Cronobacter genus using MLST, CRISPR-cas array and capsular profiling. Front Microbiol 8:1875. doi:10.3389/fmicb.2017.01875 29033918 PMC5626840

[49] Alsonosi AM, Holy O, Forsythe SJ. 2019. Characterization of the pathogenicity of clinical Cronobacter malonaticus strains based on the tissue culture investigations. Antonie van Leeuwenhoek 112:435–450. doi:10.1007/s10482-018-1178-6 30315374

[50] Forsythe SJ, Dickins B, Jolley KA. 2014. Cronobacter, the emergent bacterial pathogen Enterobacter sakazakii comes of age; MLST and whole genome sequence analysis. BMC Genomics 15:1121. doi:10.1186/1471-2164-15-1121 25515150 PMC4377842

[51] Henry M, Fouladkhah A. 2019. Outbreak history, biofilm formation, and preventive measures for control of Cronobacter sakazakii in infant formula and infant care settings. Microorganisms 7:77. doi:10.3390/microorganisms7030077 30870985 PMC6463179

[52] Osaili T, Forsythe SJ. 2009. Desiccation resistance and persistence of Cronobacter species in infant formula. Int J Food Microbiol 136:214–220. doi:10.1016/j.ijfoodmicro.2009.08.006 19720413

[53] Arku B, Fanning S, Jordan K. 2011. Heat adaptation and survival of Cronobacter spp. (formerly Enterobacter sakazakii). Foodborne Pathog Dis 8:975–981. doi:10.1089/fpd.2010.0819 21542776

[54] Walsh D, Molloy C, Iversen C, Carroll J, Cagney C, Fanning S, Duffy G. 2011. Survival characteristics of environmental and clinically derived strains of Cronobacter sakazakii in infant milk formula (IMF) and ingredients. J Appl Microbiol 110:697–703. doi:10.1111/j.1365-2672.2010.04921.x 21255207

[55] Álvarez-Ordóñez A, Begley M, Hill C. 2012. Polymorphisms in rpoS and stress tolerance heterogeneity in natural isolates of Cronobacter sakazakii. Appl Environ Microbiol 78:3975–3984. doi:10.1128/AEM.07835-11 22447602 PMC3346413

[56] Fakruddin M, Rahaman M, Ahmed MM, Hoque MM. 2014. Stress tolerant virulent strains of Cronobacter sakazakii from food. Biol Res 47:63. doi:10.1186/0717-6287-47-63 25723712 PMC4335510

[57] Jang H, Gopinath GR, Eshwar A, Srikumar S, Nguyen S, Gangiredla J, Patel IR, Finkelstein SB, Negrete F, Woo J, Lee Y, Fanning S, Stephan R, Tall BD, Lehner A. 2020. The secretion of toxins and other exoproteins of Cronobacter: role in virulence, adaption, and persistence. Microorganisms 8:229. doi:10.3390/microorganisms8020229 32046365 PMC7074816

[5] Moravkova M, Verbikova V, Huvarova V, Babak V, Cahlikova H, Karpiskova R, Kralik P. 2018. Occurrence of Cronobacter spp. in ready‐to‐eat vegetable products, frozen vegetables, and sprouts examined using cultivation and real‐time PCR methods. J Food Sci 83:3054–3058. doi:10.1111/1750-3841.14399 30468252

[59] Ling N, Li C, Zhang J, Wu Q, Zeng H, He W, Ye Y, Wang J, Ding Y, Chen M, Xue L, Ye Q, Guo W. 2018. Prevalence and molecular and antimicrobial characteristics of Cronobacter spp. isolated from raw vegetables in China. Front Microbiol 9:1149. doi:10.3389/fmicb.2018.01149 29922254 PMC5996200

[60] Ling N, Jiang X, Forsythe S, Zhang D, Shen Y, Ding Y, Wang J, Zhang J, Wu Q, Ye Y. 2022. Food safety risks and contributing factors of Cronobacter spp. Engineering 12:128–138. doi:10.1016/j.eng.2021.03.021

[61] Kim K, Jang SS, Kim SK, Park JH, Heu S, Ryu S. 2008. Prevalence and genetic diversity of Enterobacter sakazakii in ingredients of infant foods. Int J Food Microbiol 122:196–203. doi:10.1016/j.ijfoodmicro.2007.11.072 18177966

[62] Jaradat ZW, Ababneh QO, Saadoun IM, Samara NA, Rashdan AM. 2009. Isolation of Cronobacter spp. (formerly Enterobacter sakazakii) from infant food, herbs and environmental samples and the subsequent identification and confirmation of the isolates using biochemical, chromogenic assays, PCR and 16S rRNA sequencing. BMC Microbiol 9:225. doi:10.1186/1471-2180-9-225 19860874 PMC2779193

[63] Baumgartner A, Grand M, Liniger M, Iversen C. 2009. Detection and frequency of Cronobacter spp. (Enterobacter sakazakii) in different categories of ready-to-eat foods other than infant formula. Int J Food Microbiol 136:189–192. doi:10.1016/j.ijfoodmicro.2009.04.009 19419789

[64] Turcovský I, Kuniková K, Drahovská H, Kaclíková E. 2011. Biochemical and molecular characterization of Cronobacter spp. (formerly Enterobacter sakazakii) isolated from foods. Antonie van Leeuwenhoek 99:257–269. doi:10.1007/s10482-010-9484-7 20640509

[65] Hochel I, Růžičková H, Krásný L, Demnerová K. 2012. Occurrence of Cronobacter spp. in retail foods. J Appl Microbiol 112:1257–1265. doi:10.1111/j.1365-2672.2012.05292.x 22443682

[66] Li Y, Chen Q, Zhao J, Jiang H, Lu F, Bie X, Lu Z. 2014. Isolation, identification and antimicrobial resistance of Cronobacter spp. isolated from various foods in China. Food Control 37:109–114. doi:10.1016/j.foodcont.2013.09.017

[67] Singh N, Goel G, Raghav M. 2015. Prevalence and characterization of Cronobacter spp. from various foods, medicinal plants, and environmental samples. Curr Microbiol 71:31–38. doi:10.1007/s00284-015-0816-8 25855303

[68] Vojkovska H, Karpiskova R, Orieskova M, Drahovska H. 2016. Characterization of Cronobacter spp.isolated from food of plant origin and environmental samples collected from farms and from supermarkets in the Czech Republic. Int J Food Microbiol 217:130–136. doi:10.1016/j.ijfoodmicro.2015.10.017 26513253

[69] Brandão MLL, Umeda NS, Jackson E, Forsythe SJ, de Filippis I. 2017. Isolation, molecular and phenotypic characterization, and antibiotic susceptibility of Cronobacter spp. from Brazilian retail foods. Food Microbiol 63:129–138. doi:10.1016/j.fm.2016.11.011 28040160

[70] Miranda N, Banerjee P, Simpson S, Kerdahi K, Sulaiman IM. 2017. Molecular surveillance of Cronobacter spp. isolated from a wide variety of foods from 44 different countries by sequence typing of 16S rRNA, rpoB and O-antigen genes. Foods 6:36. doi:10.3390/foods6050036 28492472 PMC5447912

[71] Li Q, Li C, Chen L, Cai Z, Wu S, Gu Q, Zhang Y, Wei X, Zhang J, Yang X, Zhang S, Ye Q, Wu Q. 2023. Cronobacter spp. isolated from quick-frozen foods in China: incidence, genetic characteristics, and antibiotic resistance. Foods 12:3019. doi:10.3390/foods12163019 37628018 PMC10453260

[72] Chap J, Jackson P, Siqueira R, Gaspar N, Quintas C, Park J, Osaili T, Shaker R, Jaradat Z, Hartantyo SHP, Abdullah Sani N, Estuningsih S, Forsythe SJ. 2009. International survey of Cronobacter sakazakii and other Cronobacter Spp. in follow up formulas and infant foods. Int J Food Microbiol 136:185–188. doi:10.1016/j.ijfoodmicro.2009.08.005 19729216

[73] Pei XY, Yan L, Zhu JH, Li N, Guo YC, Fu P, Jia HY, Zhang XL, Yang XR, Yang DJ. 2016. The survey of Cronobacter spp. (formerly Enterbacter sakazakii) in infant and follow-up powdered formula in China in 2012. Biomed Environ Sci 29:99–106. doi:10.3967/bes2016.011 27003167

[74] Muytjens HL, Zanen HC, Sonderkamp HJ, Kollée LA, Wachsmuth IK, Farmer JJ 3rd. 1983. Analysis of eight cases of neonatal meningitis and sepsis due to Enterobacter sakazakii. J Clin Microbiol 18:115–120. doi:10.1128/jcm.18.1.115-120.1983 6885983 PMC270753

[75] Parra-Flores J, Holý O, Acuña S, Lepuschitz S, Pietzka A, Contreras-Fernández A, Chavarría-Sepulveda P, Cruz-Córdova A, Xicohtencatl-Cortes J, Mancilla-Rojano J, Castillo A, Ruppitsch W, Forsythe S. 2022. Genomic characterization of Cronobacter spp. and Salmonella spp. strains isolated from powdered infant formula in Chile. Front Microbiol 13:884721. doi:10.3389/fmicb.2022.884721 35722296 PMC9201451

