# Supplementary material for: Microbiological survey and genomic analysis of Cronobacter sakazakii strains isolated from US households and retail foods
Source: Appl Environ Microbiol. 2024 Jul 2;90(7):e00700-24. doi: 10.1128/aem.00700-24 (PMC11267904; doi:10.1128/aem.00700-24)
Supplement: Supplemental material — NCBI BioSample ID list. [file aem.00700-24-s0001.docx]

**Supplemental Material:**

NCBI Biosample IDs for the assembled WGS deposited at NCBI: SAMN38366867, SAMN38366868, SAMN38366869, SAMN38366870, SAMN38366871, SAMN38366872, SAMN38366873, SAMN38366874, SAMN38366958, SAMN38366959, SAMN38366960, SAMN38366961, SAMN38366962, SAMN38366963, SAMN38366966, SAMN38366967, SAMN38366968, SAMN38366969, SAMN38366995, SAMN38366996, SAMN38366997, SAMN38366998, SAMN38366999, SAMN38367000, SAMN38367001, SAMN38367002, SAMN38367004, SAMN38367005, SAMN38367723, SAMN38367724, SAMN38367966, SAMN38367967, SAMN38367968, SAMN38367969, SAMN38367970, SAMN38367997, SAMN38367998, SAMN38367999, SAMN38368000, SAMN38368001, SAMN38368002, SAMN38368003, SAMN38368004, SAMN38368005, SAMN38368006, SAMN38368007, SAMN38368008, SAMN38368009, SAMN38368896, SAMN38368897, SAMN38368898, SAMN38368899, SAMN38368900, SAMN38368901, SAMN38368902, SAMN38368903, SAMN38368904, SAMN38368937, SAMN38368938, SAMN38368939, SAMN38368940, SAMN38368941, SAMN38368942, SAMN38368943, SAMN38368944, SAMN38368945, SAMN38368946, SAMN38369330, SAMN38369331, SAMN38369332, SAMN38369333, SAMN38369334, SAMN38369335, SAMN38369336, SAMN38369337, SAMN38369338, SAMN38369339, SAMN38369341, SAMN38369342, SAMN38369345, SAMN38369352, SAMN38369354, SAMN38369355, SAMN38369356, SAMN38369357, SAMN38369358, SAMN38369416, SAMN38369417, SAMN38369418, SAMN38369419, SAMN38369420, SAMN38369421, SAMN38369422, SAMN38369423, SAMN38369476, SAMN38369477, SAMN38369478, SAMN38369479, SAMN38369480, SAMN38369481, SAMN38369482, SAMN38369498, SAMN38369507, SAMN38369508, SAMN38369509, SAMN38369510, SAMN38369511, SAMN38369512, SAMN38369513, SAMN38369514, SAMN38369585, SAMN38369586, SAMN38369587, SAMN38369588, SAMN38369589, SAMN38369590, SAMN38369591, SAMN38369594, SAMN38369595, SAMN38369596, SAMN38369634, SAMN38369635, SAMN38369636, SAMN38369637, SAMN38369638, SAMN38369639, SAMN38369640, SAMN38369803, SAMN38369804, SAMN38369805, SAMN38369806, SAMN38369807, SAMN38369808, SAMN38369886, SAMN38369887, SAMN38369888, SAMN38369890, SAMN38369891, SAMN38369892, SAMN38369893, SAMN38369894, SAMN38369895, SAMN38369896, SAMN38369897, SAMN38369898, SAMN38369899, SAMN38369900, SAMN38369928, SAMN38369929, SAMN38369952, SAMN38369953, SAMN38369954, SAMN38369955, SAMN38369956, SAMN38369957, SAMN38369958, SAMN38369959, SAMN38370102, SAMN38370104, SAMN38370109, SAMN38370112, SAMN38370114, SAMN38370116, SAMN38370120, SAMN38370121, SAMN38370224, SAMN38370226, SAMN38370249, SAMN38370255, SAMN38370257, SAMN38370260, SAMN38370272, SAMN38370273, SAMN38370274, SAMN38370275, SAMN38370276, SAMN38370277, SAMN38370291, SAMN38370292, SAMN38370293, SAMN38370294, SAMN38370295, SAMN38370296, SAMN38370297, SAMN38370298, SAMN38370299, SAMN38370300, SAMN38370301, SAMN38370316, SAMN38370317, SAMN38370318, SAMN38370319, SAMN38370320, SAMN38370382, SAMN38370383, SAMN38370384, SAMN38370385, SAMN38370386, SAMN38370387, SAMN38370388, SAMN38370389, SAMN38370390, SAMN38370391, SAMN38370392, SAMN38370393, SAMN38370394, SAMN38370395, SAMN38370618, SAMN38370620, SAMN38370621, SAMN38370668, SAMN38370669, SAMN38370670, SAMN38370671, SAMN38370672, SAMN38370673, SAMN38371680, SAMN38371681, SAMN38371682, SAMN38371683, SAMN38371684, SAMN38371685, SAMN38371686, SAMN38371687, SAMN38371708, SAMN38371710, SAMN38371712, SAMN38371716, SAMN38371718, SAMN38371719, SAMN38371720, SAMN38371721, SAMN38371722, SAMN38372145, SAMN38372146, SAMN38372147, SAMN38372148, SAMN38372149, SAMN38372150, SAMN38372151, SAMN38372152, SAMN38372153, SAMN38372174, SAMN38372176, SAMN38372178, SAMN38372180, SAMN38372182, SAMN38372184, SAMN38372187, SAMN38372193, SAMN38372195, SAMN38372199, SAMN38372200, SAMN38372201, SAMN38372217, SAMN38372218, SAMN38372219, SAMN38372221, SAMN38372222, SAMN38372223, SAMN38372224, SAMN38372225, SAMN38372226, SAMN38372234, SAMN38372237, SAMN38372240, SAMN38372243, SAMN38372245, SAMN38372246, SAMN38372249, SAMN38372251, SAMN38372366, SAMN38372368, SAMN38372369, SAMN38372371, SAMN38372372, SAMN38372373, SAMN38372374, SAMN38372380, SAMN38372382, SAMN38372383, SAMN38372385, SAMN38447608, SAMN38447609, SAMN38447610, SAMN38447611, SAMN38447612, SAMN38447613, SAMN38447614, SAMN38447615, SAMN38447616, SAMN38447617, SAMN38447618, SAMN38447639, SAMN38447640, SAMN38447641, SAMN38447647, SAMN38447648, SAMN38447649, SAMN38447650, SAMN38447651, SAMN38447652, SAMN38447653, SAMN38447654, SAMN38447671, SAMN38447672, SAMN38447673, SAMN38447674, SAMN38447675, SAMN38447676, SAMN38447677, SAMN38447678, SAMN38447679, SAMN38447680, SAMN38447681, SAMN38447710, SAMN38447711, SAMN38447712, SAMN38447713, SAMN38447714, SAMN38447715, SAMN38447716, SAMN38447717, SAMN38447718, SAMN38447719, SAMN38447720, SAMN38447732, SAMN38447733, SAMN38447735, SAMN38447736, SAMN38447737, SAMN38447740, SAMN38447741, SAMN38447773, SAMN38447774, SAMN38447775, SAMN38447777, SAMN38447778, SAMN38447802, SAMN38447804, SAMN38447806, SAMN38447807, SAMN38447808, SAMN38447809, SAMN38447811, SAMN38447814, SAMN38447823, SAMN38447824, SAMN38447825, SAMN38447826, SAMN38447827, SAMN38447828, SAMN38447829, SAMN38447830, SAMN38447832, SAMN38447833, SAMN38447856, SAMN38447857, SAMN38447858, SAMN38447859, SAMN38447863, SAMN38447864, SAMN38447865, SAMN38447866, SAMN38447868, SAMN38447869, SAMN38447870, SAMN38447871, SAMN38447874, SAMN38447875, SAMN38447876, SAMN38447877, SAMN38447878, SAMN38447899, SAMN38447905, SAMN38447910, SAMN38447912, SAMN38447914, SAMN38447915, SAMN38447916, SAMN38447938, SAMN38447957, SAMN38447958, SAMN38693319, SAMN38693330. SAMN38366867, SAMN38366868, SAMN38366869, SAMN38366870, SAMN38366871, SAMN38366872, SAMN38366873, SAMN38366874, SAMN38366958, SAMN38366959, SAMN38366960, SAMN38366961, SAMN38366962, SAMN38366963, SAMN38366966, SAMN38366967, SAMN38366968, SAMN38366969, SAMN38366995, SAMN38366996, SAMN38366997, SAMN38366998, SAMN38366999, SAMN38367000, SAMN38367001, SAMN38367002, SAMN38367004, SAMN38367005, SAMN38367723, SAMN38367724, SAMN38367966, SAMN38367967, SAMN38367968, SAMN38367969, SAMN38367970, SAMN38367997, SAMN38367998, SAMN38367999, SAMN38368000, SAMN38368001, SAMN38368002, SAMN38368003, SAMN38368004, SAMN38368005, SAMN38368006, SAMN38368007, SAMN38368008, SAMN38368009, SAMN38368896, SAMN38368897, SAMN38368898, SAMN38368899, SAMN38368900, SAMN38368901, SAMN38368902, SAMN38368903, SAMN38368904, SAMN38368937, SAMN38368938, SAMN38368939, SAMN38368940, SAMN38368941, SAMN38368942, SAMN38368943, SAMN38368944, SAMN38368945, SAMN38368946, SAMN38369330, SAMN38369331, SAMN38369332, SAMN38369333, SAMN38369334, SAMN38369335, SAMN38369336, SAMN38369337, SAMN38369338, SAMN38369339, SAMN38369341, SAMN38369342, SAMN38369345, SAMN38369352, SAMN38369354, SAMN38369355, SAMN38369356, SAMN38369357, SAMN38369358, SAMN38369416, SAMN38369417, SAMN38369418, SAMN38369419, SAMN38369420, SAMN38369421, SAMN38369422, SAMN38369423, SAMN38369476, SAMN38369477, SAMN38369478, SAMN38369479, SAMN38369480, SAMN38369481, SAMN38369482, SAMN38369498, SAMN38369507, SAMN38369508, SAMN38369509, SAMN38369510, SAMN38369511, SAMN38369512, SAMN38369513, SAMN38369514, SAMN38369585, SAMN38369586, SAMN38369587, SAMN38369588, SAMN38369589, SAMN38369590, SAMN38369591, SAMN38369594, SAMN38369595, SAMN38369596, SAMN38369634, SAMN38369635, SAMN38369636, SAMN38369637, SAMN38369638, SAMN38369639, SAMN38369640, SAMN38369803, SAMN38369804, SAMN38369805, SAMN38369806, SAMN38369807, SAMN38369808, SAMN38369886, SAMN38369887, SAMN38369888, SAMN38369890, SAMN38369891, SAMN38369892, SAMN38369893, SAMN38369894, SAMN38369895, SAMN38369896, SAMN38369897, SAMN38369898, SAMN38369899, SAMN38369900, SAMN38369928, SAMN38369929, SAMN38369952, SAMN38369953, SAMN38369954, SAMN38369955, SAMN38369956, SAMN38369957, SAMN38369958, SAMN38369959, SAMN38370102, SAMN38370104, SAMN38370109, SAMN38370112, SAMN38370114, SAMN38370116, SAMN38370120, SAMN38370121, SAMN38370224, SAMN38370226, SAMN38370249, SAMN38370255, SAMN38370257, SAMN38370260, SAMN38370272, SAMN38370273, SAMN38370274, SAMN38370275, SAMN38370276, SAMN38370277, SAMN38370291, SAMN38370292, SAMN38370293, SAMN38370294, SAMN38370295, SAMN38370296, SAMN38370297, SAMN38370298, SAMN38370299, SAMN38370300, SAMN38370301, SAMN38370316, SAMN38370317, SAMN38370318, SAMN38370319, SAMN38370320, SAMN38370382, SAMN38370383, SAMN38370384, SAMN38370385, SAMN38370386, SAMN38370387, SAMN38370388, SAMN38370389, SAMN38370390, SAMN38370391, SAMN38370392, SAMN38370393, SAMN38370394, SAMN38370395, SAMN38370618, SAMN38370620, SAMN38370621, SAMN38370668, SAMN38370669, SAMN38370670, SAMN38370671, SAMN38370672, SAMN38370673, SAMN38371680, SAMN38371681, SAMN38371682, SAMN38371683, SAMN38371684, SAMN38371685, SAMN38371686, SAMN38371687, SAMN38371708, SAMN38371710, SAMN38371712, SAMN38371716, SAMN38371718, SAMN38371719, SAMN38371720, SAMN38371721, SAMN38371722, SAMN38372145, SAMN38372146, SAMN38372147, SAMN38372148, SAMN38372149, SAMN38372150, SAMN38372151, SAMN38372152, SAMN38372153, SAMN38372174, SAMN38372176, SAMN38372178, SAMN38372180, SAMN38372182, SAMN38372184, SAMN38372187, SAMN38372193, SAMN38372195, SAMN38372199, SAMN38372200, SAMN38372201, SAMN38372217, SAMN38372218, SAMN38372219, SAMN38372221, SAMN38372222, SAMN38372223, SAMN38372224, SAMN38372225, SAMN38372226, SAMN38372234, SAMN38372237, SAMN38372240, SAMN38372243, SAMN38372245, SAMN38372246, SAMN38372249, SAMN38372251, SAMN38372366, SAMN38372368, SAMN38372369, SAMN38372371, SAMN38372372, SAMN38372373, SAMN38372374, SAMN38372380, SAMN38372382, SAMN38372383, SAMN38372385, SAMN38447608, SAMN38447609, SAMN38447610, SAMN38447611, SAMN38447612, SAMN38447613, SAMN38447614, SAMN38447615, SAMN38447616, SAMN38447617, SAMN38447618, SAMN38447639, SAMN38447640, SAMN38447641, SAMN38447647, SAMN38447648, SAMN38447649, SAMN38447650, SAMN38447651, SAMN38447652, SAMN38447653, SAMN38447654, SAMN38447671, SAMN38447672, SAMN38447673, SAMN38447674, SAMN38447675, SAMN38447676, SAMN38447677, SAMN38447678, SAMN38447679, SAMN38447680, SAMN38447681, SAMN38447710, SAMN38447711, SAMN38447712, SAMN38447713, SAMN38447714, SAMN38447715, SAMN38447716, SAMN38447717, SAMN38447718, SAMN38447719, SAMN38447720, SAMN38447732, SAMN38447733, SAMN38447735, SAMN38447736, SAMN38447737, SAMN38447740, SAMN38447741, SAMN38447773, SAMN38447774, SAMN38447775, SAMN38447777, SAMN38447778, SAMN38447802, SAMN38447804, SAMN38447806, SAMN38447807, SAMN38447808, SAMN38447809, SAMN38447811, SAMN38447814, SAMN38447823, SAMN38447824, SAMN38447825, SAMN38447826, SAMN38447827, SAMN38447828, SAMN38447829, SAMN38447830, SAMN38447832, SAMN38447833, SAMN38447856, SAMN38447857, SAMN38447858, SAMN38447859, SAMN38447863, SAMN38447864, SAMN38447865, SAMN38447866, SAMN38447868, SAMN38447869, SAMN38447870, SAMN38447871, SAMN38447874, SAMN38447875, SAMN38447876, SAMN38447877, SAMN38447878, SAMN38447899, SAMN38447905, SAMN38447910, SAMN38447912, SAMN38447914, SAMN38447915, SAMN38447916, SAMN38447938, SAMN38447957, SAMN38447958, SAMN38693319, SAMN38693330
